# Update of statistical analysis plan for: Integration of smoking cessation into standard treatment for patients receiving opioid agonist therapy who are smoking tobacco: protocol for a randomised controlled trial (ATLAS4LAR)

**DOI:** 10.1186/s13063-023-07894-w

**Published:** 2024-01-06

**Authors:** Karl Trygve Druckrey-Fiskaaen, Tesfaye Madebo, Jan Tore Daltveit, Jørn Henrik Vold, Einar Furulund, Torgeir Gilje Lid, Lars Thore Fadnes

**Affiliations:** 1 Department of Addiction Medicine, Bergen Addiction Research, Haukeland University Hospital, Bergen, Norway; 2 Department of Global Public Health and Primary Care, University of Bergen, Bergen, Norway; 3 Department of Respiratory Medicine, Stavanger University Hospital, Stavanger, Norway; 4 Division of Psychiatry, Haukeland University Hospital, Bergen, Norway; 5 Centre for Alcohol and Drug Research, Stavanger University Hospital, Stavanger, Norway; 6 Department of Clinical Science, University of Bergen, Bergen, Norway; 7 Department of Public Health, University of Stavanger, Stavanger, Norway

**Keywords:** Statistical analysis plan, Randomised controlled trial, Smoking cessation, Opioid agonist treatment

## Abstract

This protocol paper presents an updated statistical analysis plan of the protocol of a randomised controlled trial. The randomised controlled trial investigates the effect of integrating smoking cessation interventions at outpatient opioid agonist therapy (OAT) clinics for persons with opioid dependency receiving OAT medication. The intervention group receives weekly follow-up including a short behavioural intervention and provision of nicotine replacement products. The control group receives standard treatment. The duration of the intervention is 16 weeks and the follow-up was completed by the end of October 2023. The primary outcome is defined as the proportion of participants reducing the number of cigarettes smoked by at least a 50% at week 16 of the intervention period. The primary outcome will be analysed according to intention-to-treat principles. Missing outcome data will be set equal to the baseline values. Development and reporting of the statistical analysis plan follow the Guidelines for the Content of Statistical Analysis Plans in Clinical Trials.

**Trial registration** ClinicalTrials.gov NCT05290025. Registered on 22 March 2022.

**Supplementary Information:**

The online version contains supplementary material available at 10.1186/s13063-023-07894-w.

## Administrative information

**Table:** 

SAP version number with dates	Version 2, 27.09.2023
Reference to version of protocol being used	Version 2, 14.07.2022
SAP revision history	Version 1 provided in protocol from 14.07.2022
Justification for each SAP revision	Version 2 contains more detail to comply with SAP checklist [1]
Timing of SAP revisions in relation to interim analyses, etc	No interim analysis completed. Version 2 of SAP published ahead of completion of follow-up
Names, affiliations, and roles of SAP contributors	Karl Trygve Druckrey-Fiskaaen^1,2*^, Tesfaye Madebo^1,3,6^,Jan Tore Daltveit^1^, Jørn Henrik Vold^1,2,4^, Einar Furulund^1,2,5^,Torgeir Gilje Lid^5,7^, Lars Thore Fadnes^1,2^ ^1^ Bergen Addiction Research, Department of Addiction Medicine, Haukeland University Hospital, Bergen, Norway ^2^ Department of Global Public Health and Primary Care, University of Bergen, Norway ^3^ Department of Respiratory Medicine, Stavanger University Hospital, Stavanger, Norway ^4^ Division of Psychiatry, Haukeland University Hospital, Bergen, Norway ^5^ Centre for Alcohol and Drug Research, Stavanger University Hospital, Stavanger, Norway ^6^ Department of Clinical Science, University of Bergen, Norway ^7^ Department of Public Health, University of Stavanger, Norway
Person writing the SAP	Karl Trygve Druckrey-Fiskaaen
Senior statistician/analyst responsible	Jørn Henrik Vold
Chief investigator/clinical lead	Lars Thore Fadnes

## Introduction

### Background and rationale

About 85% of patients receiving opioid agonist therapy (OAT) for opioid dependence smoke tobacco [2]. Although smoke-related pulmonary diseases are significant contributors to morbidity and mortality, few smoking cessation interventions are evaluated within this group [3], and few OAT patients are offered smoking cessation as an integrated part of their addiction treatment [4]. The integration of hepatitis C virus treatment at OAT clinics improved the time to treatment initiation and the rate of sustained virological response [5]. This trial aimed to investigate whether a similar effect can be seen for the integration of smoking cessation therapy [6]. More specifically the trial aims to investigate the effect of a combined smoking cessation intervention administered weekly for up to 16 weeks on smoking patterns, psychological well-being, and physical tests. See the protocol article for further detail [6].

### Objectives

The primary objective is to assess the effect of integrating smoking cessation therapy at OAT clinics compared with standard OAT (control arm). Smoking cessation is measured by carbon monoxide levels in the exhaled air and the self-reported number of cigarettes smoked.

The secondary objectives are to investigate the change in psychological distress, impact of smoking cessation on inflammation, physical tests, and assessment of changes in quality of life, fatigue, and psychological well-being in the trial arms. The secondary objectives are specified in more detail in the published study protocol [6].

The study protocol for the randomised controlled trial on integrated smoking cessation treatment for patients who receive OAT was published in August 2022 [6]. The process of recruitment and inclusion lasted longer than anticipated at the time of publication. Recruitment was completed in July 2023. Follow-up was completed by the end of October 2023. In preparing for the analysis and publication of the primary outcomes of the trial there was a need to update and expand the statistical analysis plan included in the protocol [6]. We have used the Guidelines for the Content of Statistical Analysis Plans in Clinical Trials to guide the update [1]. The Statistical Analysis Plan (SAP) Checklist v 1.0 2019 is provided in Supplementary file 1.

## Study methods

The study is designed as a multicentre individually randomised controlled superiority trial with two parallel groups and an allocation ratio of 1:1.

The sample size calculations are provided in the protocol [6]. Based on the calculations, 133 persons were required in intervention arm and 133 persons in the control arm.

No interim analyses were planned (see details in Sect. 21b in the protocol [6]), and thus, no adjustments to significance levels were assessed.

The primary outcomes will be analysed collectively upon completion of follow-up.

## Statistical principles

All tests will be two-sided. Descriptive results and efficacy estimates will be presented with 95% confidence intervals. The statistical significance was set at *p* < 0.05.

There is only one primary outcome and thus no need to correct for multiplicity.

Adherence is defined as at least 50% attendance at weekly appointments. The total duration possible is 16 weeks.

Adherence will be presented as a histogram of the proportion of participants in the intervention each week from 0 to 16. A supplementary table will provide the number of trial participants who attended which percentage of study visits.

**Fig. 1:**
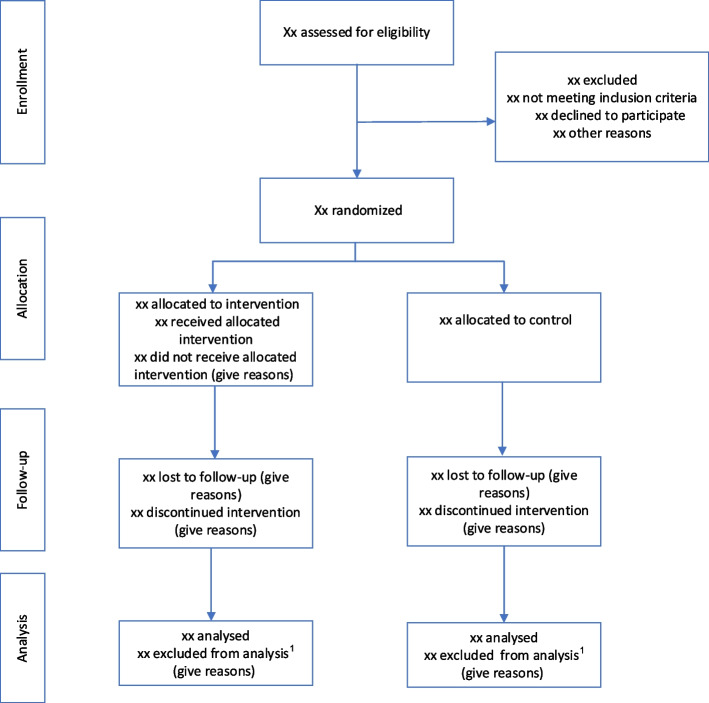
CONSORT Flow diagram. “1” indicates the following: persons who died between the date of randomization and the date of outcome evaluation (weeks 12–16) will not be included in the ITT/PP analyses but presented in the trial profile and assessed for potential severe adverse eventsable

According to Fig. 1 of the protocol, the participants were recommended a timeline for nicotine replacement therapy [6]. We will record deviations from the timeline, for example not reducing the dose of nicotine replacement products according to the plan.

We will analyse the outcomes with an intention to treat and per protocol approach. See Table 2 for more detail.

## Trial population

Figure 1 shows the information to be included in the CONSORT flow-diagram.

The withdrawals/lost to follow up will be handled according to the following principles:

- The week and, if provided by the participant, the reason for withdrawal will be noted. According to the ethics approval (no. 155386/REK Sør-øst-B, dated 23 September 2020/03 December 2021/05 April 2022) participants are allowed to withdraw without giving reasons.
- A histogram will be produced showing the number of withdrawals and loss to follow-up for each week of the trial.
- The proportion of withdrawal and loss to follow-up for the trial in total will be calculated as the sum of withdrawals and loss to follow-up each week divided by the number of persons allocated to each arm of the trial.

Table 1 indicates the baseline characteristics and how they will be summarised.

**Table 1:** Baseline characteristics and summary statistics

	Intervention	Control
Males *n* (%)		
Females *n* (%)		
Age median (IQR^a^)		
Education < 10 years *n* (%)		
Education 10–12 years *n* (%)		
Education > 12 years *n* (%)		
Homelessness *n* (%)		
Social security benefits as income *n* (%)		
Formal work as income *n* (%)		
Methadone *n (%)*		
Buprenorphine* n (%)*		
Other opioid agonist treatment medications *n *(%)		
*Substance use last 30 days n (%)*		
Illicit opioids		
Alcohol		
Amphetamines or cocaine		
Benzodiazepines		
Cannabis		
I.v. drug use last 6 months		
Debut age smoking median (IQR^a^)		
Years of smoking median (IQR^a^)		
Average daily number of cigarettes smoked		
Body mass index median (IQR^a^)		
Obstructive pulmonary disease^b^ *n* (%)		

^a^Inter-quartile range

^b^Forced expiratory volume in the first second (FEV1)/forced vital capacity (FVC) ratio < 70% in spirometry

## Analysis

We will use Stata/SE17 (StataCorp, TX, USA) for the statistical analysis.

### Primary outcome

The analysis of the primary outcome measures will be completed according to intention to treat (ITT) principles (Table 2). If primary outcome data are missing, we will set these equal to baseline values for the ITT analysis. The primary outcome is defined as the proportion of participants who achieve at least a 50% reduction in the number of cigarettes smoked by week 16 of the intervention period (range 12–16 weeks after intervention initiation), including those who achieve smoking cessation. This is assessed with self-reported cigarette use and verified by carbon monoxide (CO) levels and expected to be below six parts per million (ppm) among self-reported non-smokers.

**Table 2:** Analysis and presentation of the primary outcome

Outcome	Events *n* (%)		Absolute difference between arms (*n*/%, 95%CI)	Logistic regression^a^ odds ratio (95% CI)
	Intervention	Control		
Smokers^b^ at 16 weeks, ITT^c^				
Carbon monoxide < 6 ppm, *N* (%)				
Smokers at 16 weeks, PP^d^				
At least 50% reduction number^e^ of cigarettes at 16 weeks, ITT^c^				
At least 50% reduction number^e^ of cigarettes at 16 weeks, PP^d^				
Number of cigarettes smoked/day^c^				
Severe adverse events (assumed linked)				

^a^Unadjusted analysis unless Table 1 indicate substantial differences between arms at baseline

^b^A person smoking at least one cigarette per day or seven cigarettes per week

^c^*ITT*, intention to treat population: participants assessed according to randomisation regardless of adherence to trial. Any missing data in the outcome variable will be set equal to baseline

^d^*PP*, per protocol population: all participants who completed at least 50% of the trial visits

^e^The average daily number of cigarettes smoked, as reported by the participant

A sensitivity analysis of the effect of missing data will be completed according to Table 3.

**Table 3:** Sensitivity analysis of handling missing data on the primary outcome

Outcome	Handling of missing data at end of trial	Events *n* (%)		Absolute difference between arms (*n*, 95%CI)	Logistic regression^a^ odds ratio (95% CI)
		Intervention	Control		
No. of smokers^b^ at 16 weeks, ITT^c^	Equal to baseline^d^				
	Person excluded^e^				
No. of smokers^b^ at 16 weeks, PP^f^	Equal to baseline^d^				
	Person excluded^e^				
At least 50% reduction number^g^ of cigarettes at 16 weeks, ITT	Equal to baseline^d^				
	Person excluded^e^				
At least 50% reduction number^g^ of cigarettes at 16 weeks, PP	Equal to baseline^d^				
	Person excluded^e^				

^a^Unadjusted analysis unless Table 1 indicates substantial differences between arms at baseline

^b^A person smoking at least one cigarette per day or seven cigarettes per week

^c^*ITT*, intention to treat population: participants assessed according to randomisation regardless of adherence to trial

^d^If data on primary outcome is missing at 16 weeks, the results are set equal to baseline

^e^If data on primary outcome is missing at 16 weeks the person is excluded from the analysis (complete case)

^f^ *PP*, per protocol population: all participants who completed at least 50% of the trial visits

^g^The average daily number of cigarettes smoked, as reported by the participant

The proportions of smokers in both arms at baseline and 16 weeks will be presented in a bar chart.

We will perform exploratory (hypothesis generating) subgroup analysis (presented as forest plots) of the primary outcome with the following subgroups (at baseline):

- Age: < 40, 40–60, > 60
- Sex (male/female)
- Obstructive pulmonary disease (yes/no)
- OAT medication (buprenorphine vs. methadone/other)
- Intravenous drug use (yes/no)
- Years of smoking: < 5, 5–15, > 15 (not in the original analysis plan)

### Secondary outcome

The secondary outcomes will be analysed according to Table 4 as changes from baseline (day of enrolment). Data for the secondary analysis will be collected by week 16 of the intervention (range 12–16 weeks after intervention initiation).

**Table 4:** Plan for analysis of secondary outcomes

Outcome	Hypothesis	Outcome measure	Method of analysis
Number of cigarettes smoked	Reduction in number of cigarettes	Self-reported daily number of cigarettes smoked	*t*-test and regression methods with secondary outcomes as dependent variable adjusted for variables defined in Table 2
Carbon monoxide in exhaled air	Reduced CO levels	Carbon monoxide in ppm in exhaled air	
C-reactive protein	Reduced levels	CRP in mg/L	
Leucocyte count	Levels within reference limit	Leucocyte count in 10^9^/L	
Psychological well-being	Increased score	Hopkins Symptom Checklist (SCL-10)	
Physical fitness	Increased score	4-min step test, number of steps	
Quality of life	Increased score	EuroQoL EQ-5D-5L-questionaire	
Fatigue	Less Fatigue	Fatigue Symptom Scale (FSS-3)	
Dyspnoea	Less after intervention	Modified Medical Research Council (mMRC)-scale	
Physical activity	Increased	Physical Activity Questionnaire (IPAQ)	

### Additional analysis

We will examine the validity of self-reported cigarette use by correlation and/or Spearman’s rank between the number of cigarettes smoked and smoking intensity determined by carbon monoxide in exhaled air [7].

### Methods used for assumptions to be checked for statistical methods

The participants were randomly assigned to ensure comparable intervention and control arms. The analysis methods will follow the CONSORT and SPIRIT guidelines. Categorical or continuous variables will be summarised as percentages, median with interquartile range or means with standard deviation for variables with Gaussian distribution. The outcomes will be checked for the assumptions of independent outcomes, limited influence of outliers and non-multicollinearity hold.

Potential confounders may be considered for adjustment if they are imbalanced at baseline. Missing data will be considered, and imputation based on predefined assumptions (baseline values) will be performed when necessary.

### Handling of missing data

Missing data in the outcome variables will be handled using an intention to treat strategy, i.e. the value is set equal to baseline.

### Adverse event reporting and harms

We will report the number of grade 3/4 adverse events and for each event details of the event and causality considerations.

### Supplementary Information

**Additional file 1:** **Supplementary file 1.** Statistical Analysis Plan (SAP) Checklist v 1.0 2019.

## Data Availability

The authors and persons mentioned under the “Acknowledgements” section will have access to the final trial dataset.
