# Supplementary material for: Update of statistical analysis plan for: Integration of smoking cessation into standard treatment for patients receiving opioid agonist therapy who are smoking tobacco: protocol for a randomised controlled trial (ATLAS4LAR)
Source: Trials. 2024 Jan 6;25:29. doi: 10.1186/s13063-023-07894-w (PMC10770886; doi:10.1186/s13063-023-07894-w)
Supplement: Supplementary file 1 — Additional file 1: Supplementary file 1. Statistical Analysis Plan (SAP) Checklist v 1.0 2019. [file 13063_2023_7894_MOESM1_ESM.docx]

**Statistical Analysis Plan (SAP) Checklist v 1.0 2019**

| Section/Item | Index | Description | Reported on page # |
| --- | --- | --- | --- |
| **Section 1: Administrative information** | | | |
| Trial and Trial registration | 1a | Descriptive title that matches the protocol, with SAP either as a forerunner or subtitle,  and trial acronym (if applicable) | 1 |
|  | 1b | Trial registration number | 2 |
| SAP Version | 2 | SAP version number with dates | 2 |
| Protocol Version | 3 | Reference to version of protocol being used | 2 |
| SAP revisions | 4a | SAP revision history | 2 |
|  | 4b | Justification for each SAP revision | 3 |
|  | 4c | Timing of SAP revisions in relation to interim analyses, etc. | 2 |
| Roles and responsibility | 5 | Names, affiliations, and roles of SAP contributors | 2 |
| Signatures of: | 6a | Person writing the SAP | 2 |
|  | 6b | Senior statistician responsible |  |
|  | 6c | Chief investigator/clinical lead | 2 |
| **Section 2: Introduction** | | | |
| Background and rationale | 7 | Synopsis of trial background and rationale including a brief description of research question  and brief justification for undertaking the trial | 3 |
| Objectives | 8 | Description of specific objectives or hypotheses | 3 |
| **Section 3: Study Methods** | | | |
| Trial design | 9 | Brief description of trial design including type of trial (e.g., parallel group, multi-arm, crossover, factorial)  and allocation ratio and may include brief description of interventions | 3 |
| Randomization | 10 | Randomization details, e.g., whether any minimization or stratification occurred (including stratifying  factors used or the location of that information if it is not held within the SAP) | 3 and section 16 of protocol (1) |
| Sample size | 11 | Full sample size calculation or reference to sample size calculation in protocol  (instead of replication in SAP) | In protocol section 14 (1) |
| Framework | 12 | Superiority, equivalence, or noninferiority hypothesis testing framework, including which comparisons  will be presented on this basis | 3 |
| Statistical interim analysis and stopping guidance | 13a | Information on interim analyses specifying what interim analyses will be carried out  and listing of time points | 3 |
|  | 13b | Any planned adjustment of the significance level due to interim analysis | 3 |
|  | 13c | Details of guidelines for stopping the trial early | In protocol section 22 (1) |
| Timing of final analysis | 14 | Timing of final analysis, e.g., all outcomes analysed collectively or timing stratified  by planned length of follow-up | 3 |
| Timing of outcome assessments | 15 | Time points at which the outcomes are measured including visit “windows” | 3 and table 1 of protocol (1) |
| **Section 4: Statistical Principals** | | | |
| Confidence intervals and *P* values | 16 | Level of statistical significance | 3 |
|  | 17 | Description and rationale for any adjustment for multiplicity and, if so, detailing how the type 1 error  is to be controlled | 3 |
|  | 18 | Confidence intervals to be reported | 3 |
| Adherence and Protocol deviations | 19a | Definition of adherence to the intervention and how this is assessed including extent  of exposure | 3 |
|  | 19b | Description of how adherence to the intervention will be presented | 4 |
|  | 19c | Definition of protocol deviations for the trial | Section 11b of protocol(1) |
|  | 19d | Description of which protocol deviations will be summarized | 3 |
| Analysis populations | 20 | Definition of analysis populations, e.g., intention to treat, per protocol,  complete case, safety | 4 |
| **Section 5: Trial Population** | | | |
| Screening data | 21 | Reporting of screening data (if collected) to describe representativeness  of trial sample | n.a. |
| Eligibility | 22 | Summary of eligibility criteria | Section 10 of protocol(1) |
| Recruitment | 23 | Information to be included in the CONSORT flow diagram | 8 |
| Withdrawal/ Follow-up | 24a | Level of withdrawal, e.g., from intervention and/or from follow-up | 4 and 8 |
|  | 24b | Timing of withdrawal/lost to follow-up data | 4 and 8 |
|  | 24c | Reasons and details of how withdrawal/lost to follow-up data will be presented | 4 and 8 |
| Baseline patient characteristics | 25a | List of baseline characteristics to be summarized | 4 and 5 |
|  | 25b | Details of how baseline characteristics will be descriptively summarized | 4 and 5 |
| **Section 6: Analysis** | | | |
| Outcome definitions |  | List and describe each primary and secondary outcome including details of: | 4 |
|  | 26a | Specification of outcomes and timings. If applicable include the order of importance of primary  or key secondary end points (e.g., order in which they will be tested) | 4 |
|  | 26b | Specific measurement and units (e.g., glucose control, hbA1c [mmol/mol or %]) | 7 |
|  | 26c | Any calculation or transformation used to derive the outcome (e.g., change from baseline, QoL score,  Time to event, logarithm, etc.) | 4 |
| Analysis methods | 27a | What analysis method will be used and how the treatment effects will be presented | 5 |
|  | 27b | Any adjustment for covariates | 5 |
|  | 27c | Methods used for assumptions to be checked for statistical methods |  |
|  | 27d | Details of alternative methods to be used if distributional assumptions do not hold, e.g., normality,  proportional hazards, etc. | 5 |
|  | 27e | Any planned sensitivity analyses for each outcome where applicable | 6 |
|  | 27f | Any planned subgroup analyses for each outcome including how subgroups are defined | 4 |
| Missing data | 28 | Reporting and assumptions/statistical methods to handle missing data (e.g., multiple imputation) | 5 |
| Additional analyses | 29 | Details of any additional statistical analyses required, e.g., complier-average causal effect10 analysis | n.a. |
| Harms | 30 | Sufficient detail on summarizing safety data, e.g., information on severity, expectedness, and causality;  details of how adverse events are coded or categorized; how adverse event data will be analysed,  i.e., grade 3/4 only, incidence case analysis, intervention emergent analysis | 5 |
| Statistical software | 31 | Details of statistical packages to be used to carry out analyses | 4 |
| References | 32a | References to be provided for nonstandard statistical methods | n.a. |
|  | 32b | Reference to Data Management Plan | n.a. |
|  | 32c | Reference to the Trial Master File and Statistical Master File | n.a. |
|  | 32d | Reference to other standard operating procedures or documents to be adhered to | n.a. |

**Taken from the paper:** Gamble C, Krishan A, Stocken D, Lewis S, Juszczak E, Doré C, et al. Guidelines for the Content of Statistical Analysis Plans in Clinical Trials. JAMA. 2017;318(23):2337-43.

1. Druckrey-Fiskaaen KT, Furulund E, Daltveit JT, Vold JH, Lid TG, Madebo T, et al. Integration of smoking cessation into standard treatment for patients receiving opioid agonist therapy who are smoking tobacco: protocol for a randomised controlled trial (ATLAS4LAR). Trials. 2022;23(1):663.
